# Qian lie an suppository (prostant) for chronic prostatitis: A systematic review and meta-analysis of randomized controlled trials

**DOI:** 10.1097/MD.0000000000015072

**Published:** 2019-04-05

**Authors:** Cao Hui-juan, Liang Shi-bing, Liu Jian-ping, Wang Bin, Li Hai-song, Wang Ji-sheng, Guan Si-qi, Zhu Yu-tian, Dai Heng-heng, Zhou Chun-yu

**Affiliations:** a Centre for Evidence-Based Chinese Medicine, Beijing University of Chinese Medicine, Beijing; b Graduate school, Shanxi University of Chinese Medicine, Taiyuan, Shanxi Province, China; c Dong Zhi Men Hospital, the 1st Affiliated Hospital of Beijing University of Chinese Medicine; d Office of Science and Technology Administration, Beijing University of Chinese Medicine, Beijing, China.

**Keywords:** chronic prostatitis, meta-analysis, prostant, Qian Lie an suppository, systematic review

## Abstract

**Background:**

Chronic prostatitis (CP) is an inflammation of the prostate gland that seriously affects the quality of life of patients. The existing evidence of antibiotics and α-blockers for the treatment of CP is limited.

**Objectives:**

This review evaluated the effectiveness and safety of Qian Lie An Suppository (Prostant) in treating CP.

**Methods:**

Randomized controlled trials comparing Prostant (alone or plus the control) with placebo, conventional drugs, or nonpharmaceutical therapies for CP were included in this article through searching from 6 databases. Data were analyzed using RevMan 5.3 software. Meta-analysis was performed when the clinical or statistical heterogeneity was found acceptable among trials. Estimate effects were present with risk ratio (RR) or mean difference and their 95% confidence interval (CI) for dichotomies or continuous variables. Quality of the evidence for each primary outcome was assessed using GRADE criteria.

**Results:**

Totally 21 trials involving 3359 participants were included. There were 2 included trials had unclear risk of bias, and the remaining trials had high risk of bias. Meta-analyses showed the number of cured patients in the Prostant group was 2 times more than that of the placebo (RR 2.05, 95%CI 1.10 to 3.81) or antibiotics (RR 1.95, 95%CI 1.18 to 3.23) groups. Similar results were found when Prostant in combination with antibiotics or hyperthermia compared with the antibiotics (RR 1.78, 95% CI 1.10–2.89) or hyperthermia (RR 1.72, 95% CI 1.23–2.40) alone. However, there was no difference in the number of cured patients between Prostant and α-blockers or hyperthermia therapy. No severe adverse event was reported in all included trials. The main adverse events in Prostant group were reported (in 8 included trials) as diarrhea and anal discomfort.

**Conclusions:**

Low-quality evidence showed that the Prostant may have add-on effect for patients with CP on increasing the number of cured patients, relieving pain, and improving the quality of life. There is not sufficient evidence to determine the effectiveness and safety of Prostant for the treatment of CP compared with placebo, antibiotics, α-blockers or the hyperthermia therapy.

## 1 Introduction

Chronic prostatitis (CP) is an inflammation of the prostate gland. Its incidence was estimated at 2.0% to 13.0% worldwide and at 4.5% to 32.9% in China.^[1–3]^ The main clinical manifestations of CP include repeated episodes of odynuria, abnormal urination (e.g., urgent or frequent urination), increased white blood cells in the prostatic fluid and so on. Patients with CP often suffer from sexual desire disorders, with hypoactive sexual desire and sexual dysfunction. CP seriously affects the quality of life of patients and is difficult to cure.

CP could be classified as chronic bacterial prostatitis (CBP) and chronic abacterial prostatitis (CAP). Both CBP and CAP are commonly treated with antibiotics and/or α-blockers. However, the use of antibiotics and/or α-blockers for CAP is not supported by the existing evidence according to a Cochrane systematic review.^[4]^ The evidence supporting the use of antibiotics and other medications for CBP are also limited.^[5,6]^

Prostant (Qian Lie An Suppository) is a traditional Chinese patent medicine commonly used in China for the treatment of CP. According to the instruction, Prostant should be placed 3 to 4 cm inside the opening of the rectum, one suppository each time, once a day. The ingredients include *Huang Bai* (Cortex Phellodendri), *Hu Zhang* (Rhizoma Polygoni Cuspidati), *Zhi Zi* (Fructus Gardeniae), *Da Huang* (Radix et Rhizoma Rhei), and so on. The main ingredient is *Huang Bai*. Pharmacological study shows that the berberine, the main component of *Huang Bai,* can inhibit the transcriptional activity of cyclooxygenase (COX-2), block the formation of inflammatory transmitters, reduce the infiltration of inflammatory cells between tissues, and adjust the immune function in patients with CP.^[7,8]^ These functions can reduce inflammation, relieve urethral resistance, and relieve symptoms of CP. A study by isotope tracer method showed that the effective component of the Prostant can reach the target organ (prostate) quickly, and the concentration in the prostate is higher than that of the other organs with the exception of the rectum, liver, kidney, and still maintains a certain level within 24 hours.^[9]^

A systematic review^[10]^ of 4 trials with 784 patients showed that the Prostant can reduce the white blood cells in the prostatic fluid [Risk Ratio (RR) 1.81, 95% confidence interval (CI) 1.29–2.53], reduce the pain (RR 1.36, 95% CI 1.14–1.62) and improve the urination symptoms (RR 2.25, 95% CI 1.51–3.37). The total effective rate of treatment was statistically significant (RR 2.36, 95%CI 1.79 to 3.10, *P < *.00001). Therefore, the authors conclude that the Prostant is effective in the treatment of CP. However, the strength of the conclusion is limited due to the low methodological quality and the small sample size of included studies. Since the previous review was published in 2006, it is worthy to update the evidence with more potential high-quality studies.

This review is aimed to investigate the effectiveness and safety of Prostant for the treatment of patients with CP in terms of dysuria, abnormal urination, quality of life and other related outcomes.

## 2 Methods

The protocol of this review was registered in PROSPERO by Huijuan Cao, Shibing Liang, Jianping Liu, Bin Wang, Haisong Li, as “Qian Lie An Shuan (Prostant) for the treatment of chronic prostatitis (CP): a systematic review of randomized controlled trials”. (ID: PROSPERO 2018 CRD42018094399, and available from: http://www.crd.york.ac.uk/PROSPERO/display_record.php?ID=CRD42018094399)

### 2.1 Criteria for the inclusion

Randomized controlled trials (RCTs) comparing Prostant with placebo, no treatment, standard treatment (including Western drugs, such as antibiotics, andα-blockers), or nonpharmaceutical therapies in treating chronic prostatitis and reporting at least one of the below outcomes were included in this review. Prostant in combination with other treatment compared with other treatment alone was also included. Chronic prostatitis should be diagnosed in accordance with a recognized criterion. The primary outcome of this review included the number of cured participants, and the improvement of clinical symptoms, which assessed by NIH-CPSI (National Institute of Health-Chronic Prostatitis Symptoms Index) scores. The secondary outcomes included the improvement of each main clinical symptoms (including pain, paruria, and/or quality of life) which were assessed through recognized quantitative evaluation methods (such as the subitems of NIH-CPSI), the improvement of sexual dysfunction, the prostatic fluid examination results [e.g., Expressed Prostatic Secretions-White blood cells (EPS-WBC)] and adverse events.

### 2.2 Searching strategy

PubMed, EMBASE, the Cochrane Library, the Chinese National Knowledge Infrastructure Databases (CNKI), the Chongqing VIP China Science and Technology Journal Database (VIP) and Wanfang Database were searched from their inception to October 30, 2018.

The subject/MeSH terms used for the searches were: “Prostant” OR “Qian Lie An Shuan (Qian Lie An Suppository)” combined with “prostatitis” OR “prostatism” OR “chronic prostatitis”, and adjusted for use in the different databases.

### 2.3 Literature screening and data extraction

Five authors (LSB, WJS, GSQ, ZYS, and DHH) screened the literatures and selected the eligible trials according to the inclusion criteria. Disagreements were solved by discussion with another author (CHJ). Data, including authors information, characteristics of participants, details of intervention and control, outcomes, and information relevant to study design, were extracted by 2 authors according to the predesigned forms.

### 2.4 Risk of bias assessment

The methodological quality of the included trials were assessed using the risk of bias tool recommended by the Cochrane Collaboration,^[11]^ in which 7 elements are assessed: random sequence generation, allocation concealment, blinding of participants and personnel, blinding of outcome assessment, incomplete outcome data (according to record the missing data and the method to deal with it), selective reporting (determined by the consistency of the predefined and reported outcomes), and other bias (assessed according to sample size calculation, inclusion/exclusion criteria for patients’ recruitment, comparability of baseline data, funding sources). Finally, we made a judgment of “low risk of bias,” “high risk of bias,” or “unclear risk of bias” for each included trial.

### 2.5 Data analysis

All statistical analyses were performed using RevMan 5.3 (The Cochrane Collaboration) software. We summarized the data using risk ratio (RR) calculations and 95% confidence intervals (CI) for binary outcomes, and mean difference (MD) with 95% CI for continuous outcomes.

Statistical heterogeneity among the included trials were evaluated using the *I*^2^ test, and a meta-analysis was conducted if there proved to be no significant clinical (relating to the participants, interventions, controls, and outcomes) and statistical heterogeneity (*I*^2^ values are less than 75%) between the included trials. If the *I*^2^ value is <25%, we used a fixed-effect model (FEM) to pool the data, and if it is between 25% and 75%, we estimated the sources of the heterogeneity first. If the statistical heterogeneity is explained successfully by sensitive analysis or subgroup analysis (i.e., *I*^2^ is <25%), we also used the FEM to pool the data, otherwise, a random-effects model (REM) was applied. Data were not pooled if there was a significant level of statistical heterogeneity among the trials (i.e., *I*^2^ is >75%) and was not possible to explain or to handle (by subgroup analysis).

Subgroup analyses was planned to conducted to determine the effects of different age group of patients (middle-aged or elderly people), the different types of patients (CBP or CAP), or of the different treatment duration (short term or long term) on the results, if the data available. Meanwhile, sensitivity analysis was also intended to be conducted to challenge the robustness of the primary analysis, for trials with/without a high risk of bias, and for meta-analyses conducted using the FEM and the REM.

In addition, a funnel plot was planned to be applied to explore the possibility of publication bias, however, since insufficient trials was included in one meta-analysis (<ten), we did not conduct the funnel plot in the end.

The GRADE (Grading of Recommendations Assessment, Development and Evaluation criteria) assessment was conducted to evaluate the quality of evidence for each primary outcome (with synthesized results). The factors that downgraded the quality include imprecision, inconsistency, indirectness, limitations, and bias of the evidence.

## 3 Results

### 3.1 Searching results

**Figure 1:**
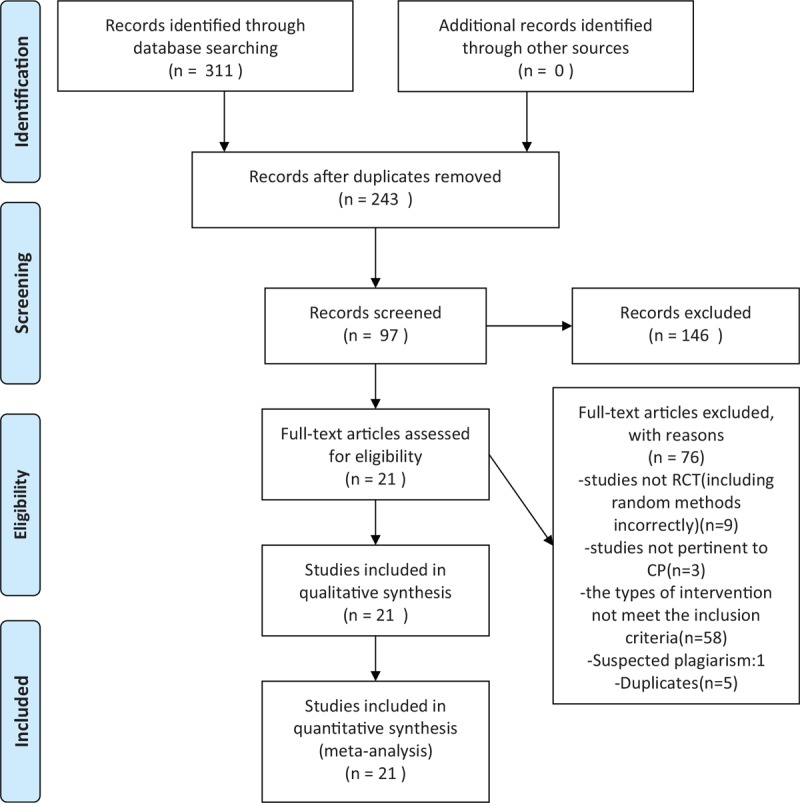
Flow diagram.

After searching from the above-mentioned 6 databases, 311 citations were found and 214 of them were screened out by reading the titles and abstracts. The full-text articles 97 citations were further reviewed and 76 of them did not meet our inclusion criteria. Finally, 21^[12–32]^ studies were included in this review. Figure 1 shows the study flowchart.

### 3.2 Characteristics of the included trials

The 21 trials were all published in Chinese. Eleven of them were 2-arm parallel controlled trials, and the remaining 10 were 3-arm trials. A total of 3359 men were included in this review, and the sample size varied from 40 to 400 among trials with an average of 63 participants per group. Three of the included trials did not report the age of participants, and other studies showed the age was between 18 to 68 years old. All of the participants were diagnosed according to recognized criteria.

All the included trials used Prostant as the main treatment, one suppository daily. The suppository was inserted into the rectum about 3 to 4 cm inside the opening of the rectum. Among them, 15 trials observed the add-on effect of Prostant, which means they compared the combination of Prostant and other therapies to other therapies alone.

There were 4 types of control: placebo (3 trials), antibiotics (10 trials), α-blockers (Tamsulosin hydrochloride or Terazosin) (4 trials), and nonpharmaceutical therapies (mainly hyperthermia therapy) (4 trials). Table 1 demonstrates the details of the comparison.

**Table 1:**
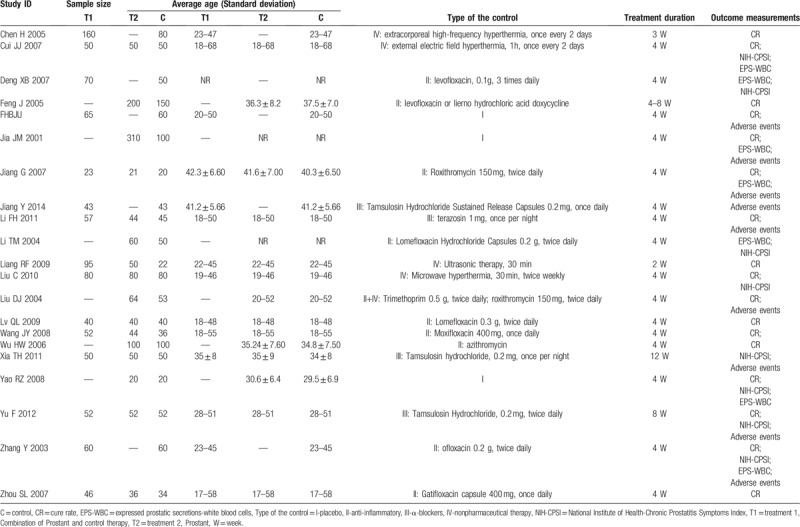
Characteristics of included 21 trials on Prostant for chronic prostatitis.

Seventeen trials reported the cure rate as the primary outcome, the total or partial of the NIH-CPSI scores were reported in 8 trials, EPS-WBC were reported in 7 trials, and adverse events were mentioned in 9 trials. None of the trial reported the improvement of sexual dysfunction.

### 3.3 Methodological quality of the included trials

According to our predefined criteria, most of the included trials had poor methodological quality. Three trials described the methods of random number generation (as using random number table), however, none of them mentioned the allocation concealment. Three trials applied placebo as control, and blinding to patients was impossible to be used in other 18 trials in which the suppository was compared with oral administration drugs or external hyperthermia therapy. Insufficient information was provided for judgement in all the 21 trials on the risk of bias from lack of blinding to assessors. Four trials were assessed as high risk of attrition bias due to the inappropriate methods on dealing with missing data. No protocol could be found for all trials, thus we assessed all of them as having unclear risk of selective reporting bias.

**Figure 2:**
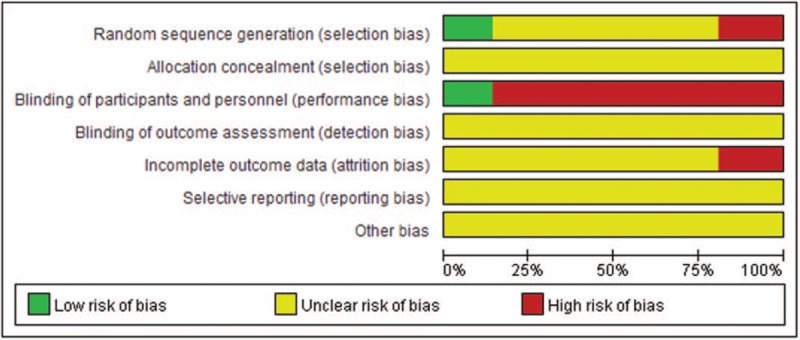
Risk of bias graph about each risk of bias item presented as percentages across all included studies.

In summary, only 2 included trials had unclear risk of bias, and the remaining 19 trials were all had high risk of bias. Figure 2 shows the risk of bias summary.

### 3.4 Estimate effects

#### 3.4.1 Prostant versus placebo

**Figure 3:**
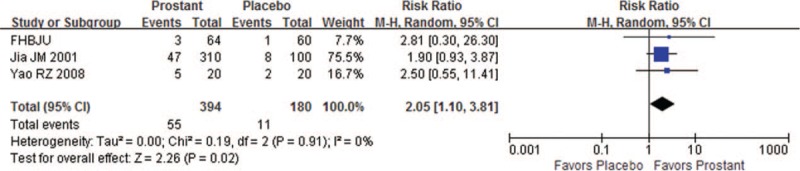
Forest plot of comparison between Prostant and placebo on number of cured participants.

Three trials compared Prostant with placebo control. Results from single studies showed no significant difference between the groups in improving NIH-CPSI scores (MD -1.15, 95%CI −4.82–2.12, 1 trial, 40 participants) and EPS-WBC scores (MD −3.90, 95%CI −10.58–2.78, 1 trial, 40 participants). However, meta-analysis of the 3 trials found more cured patients in Prostant group than that in placebo group (RR 2.05, 95%CI 1.10–3.81, 3 trials, 574 participants, *I*^*2*^=0%, see Fig. 3).

#### 3.4.2 Prostant versus drugs

Ten trials compared Prostant with drugs, 3 of them used α-blockers and the remaining 7 trials used antibiotics.

**Figure 4:**
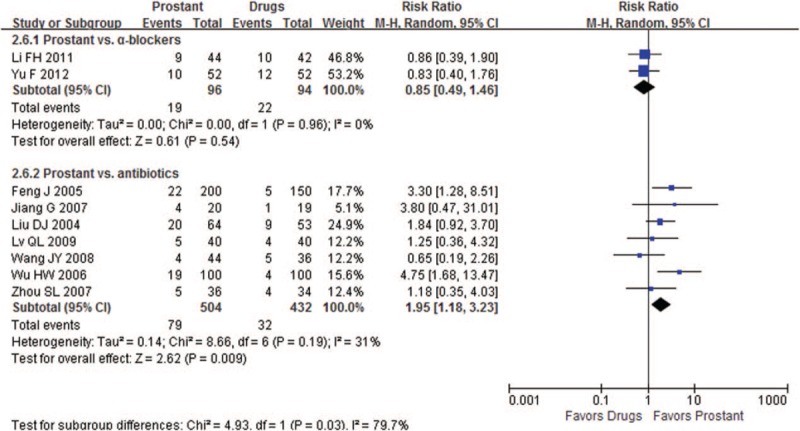
Forest plot of comparison between Prostant and drugs on number of cured participants.

Meta-analysis showed no significant difference between Prostant and α-blockers in improving symptoms according to neither the total scores of NIH-CPSI (MD -0.42, 95% CI −1.08–0.23, 2 trials, 204 participants, *I*^*2*^=0%), nor the scores of NIH-CPSI for pain intensity (MD −0.41, 95%CI −1.64–0.82, 2 trials, 204 participants, *I*^*2*^=80%), paruria (MD 0.23, 95%CI −0.52 to 0.98, 2 trials, 204 participants, *I*^*2*^=78%), quality of life (MD 0.16, 95%CI −0.28 to 0.60, 2 trials, 204 participants, *I*^*2*^=0%). Number of the cured patients were similar in 2 groups (RR 0.85, 95% CI 0.49–1.46, 2 trials, 190 participants, *I*^*2*^=0%, see Fig. 4).

When compared Prostant to antibiotics (including levofloxacin, moxifloxacin, ofloxacin, gatifloxacin, and azithromycin), either results from single study or meta-analysis found that Prostant may have better effect on decreasing the NIH-scores for quality of life (MD −1.00, 95%CI −1.90 to −0.10, 1 trial, 110 participants), the EPS-WBC scores (MD −2.00, 95%CI −2.61 to −1.39, 1 trial, 110 participants) (MD −8.10, 95%CI −9.00 to −7.20, 1 trial, 39 participants) and increasing the number of cured patients (RR1.95, 95%CI 1.18 to 3.23, 7 trials, 936 participants, *I*^*2*^=31%, see Fig. 4).

#### 3.4.3 Prostant plus drugs versus drugs

Four trials observed add-on effect of Prostant based on using α-blockers, and another 6 trials compared the combination of Prostant and antibiotics with antibiotics alone.

**Figure 5:**
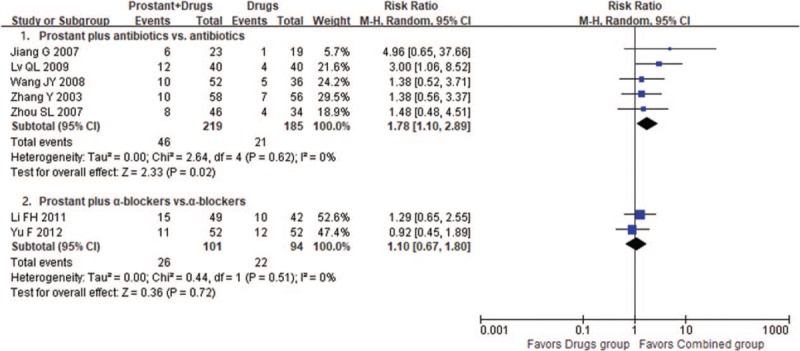
Forest plot of comparison between combination therapies and drugs on number of cured participants.

Meta-analysis of 2 trials found the combination therapy was superior in improving the symptoms of pain (MD −1.69, 95% CI −2.09 to −1.29, 2 trials, 204 participants, *I*^*2*^=11%) and paruria (MD −0.85, 95% CI −0.96 to −0.74, 2 trials, 204 participants, *I*^*2*^=0%). Single studies also found the combination therapy was superior in decreasing the total NIH-CPSI scores (MD −2.70, 95% CI −3.31 to −2.09, 1 trial, 100 participants) (MD -6.63, 95% CI -10.10 to −3.16, 1 trial, 104 participants). However, no difference was found between groups in the number of cured patients (RR1.10, 95% CI 0.67–1.80, 2 trials, 195 participants, *I*^*2*^=0%, see Fig. 5).

One trial showed Prostant had good add-on effect on decreasing total scores of NIH-CPSI (MD −3.00, 95% CI −3.91 to −2.09, 1 trial, 120 participants) compared to antibiotics alone. Results from 3 trials also found average 4.15 scores decrease of EPS-WBC scores in combination group compared with control, although the meta-analysis could not be conducted due to significant statistical heterogeneity (*I*^*2*^=99%). Meta-analysis showed the combination therapy was significantly better in increasing the number of cured patients (RR1.78, 95% CI 1.10–2.89, 5 trials, 404 participants, *I*^*2*^=0%, see Fig. 5).

#### 3.4.4 Prostant versus nonpharmaceutical therapies

**Figure 6:**
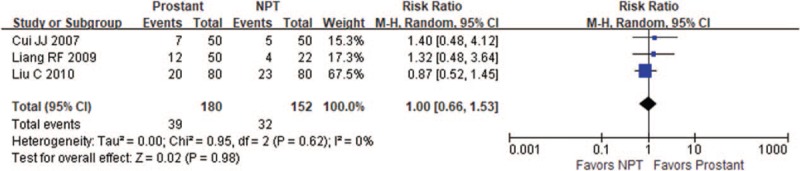
Forest plot of comparison between Prostant and nonpharmaceutical therapy on number of cured participants.

Three trials compared Prostant with external hyperthermia. The results showed the external hyperthermia was better than Prostant in decreasing the total scores of NIH-CPSI (MD 2.27, 95% CI 0.72–3.81, 2 trials, 260 participants, *I*^*2*^=37%) and EPS-WBC (MD 1.10, 95% CI 0.63−1.57, 1 trial, 100 participants). However, no significant difference was found between groups in increasing the number of cured patients (RR1.00, 95% CI 0.66−1.53, 3 trials, 332 participants, *I*^*2*^=0%, see Fig. 6).

#### 3.4.5 Prostant plus nonpharmaceutical therapies versus nonpharmaceutical therapies alone

**Figure 7:**
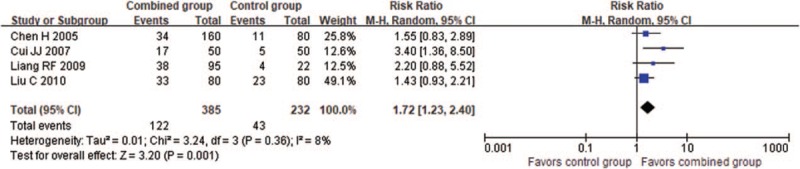
Forest plot of comparison between combination therapies and nonpharmaceutical therapy on number of cured participants.

Meta-analysis from 4 trials showed the combination of Prostant and hyperthermia therapy was better than hyperthermia alone in decreasing the total scores of NIH-CPSI (MD -3.16, 95% CI −4.24 to −2.07, 2 trials, 260 participants, *I*^*2*^=0%) and increasing the number of cured patients (RR 1.72, 95% CI 1.23−2.40, 4 trials, 617 participants, *I*^*2*^=8%, see Fig. 7).

### 3.5 Adverse events

Nine trials reported adverse events information. One trial reported that no adverse event was occurred in both groups. The remaining 8 trials reported that the average incidence rate of adverse events in Prostant group was 12.78%. They were mild- or moderate diarrhea and anal discomfort. No severe adverse event was reported in all the included trials.

## 4 Discussion

### 4.1 Summary of the main findings

This review involved 21 trials with 3359 participants. Meta-analyses showed the number of cured patients in the Prostant group was twice more than that of the placebo (RR 2.05, 95%CI 1.10−3.81) or antibiotics (RR 1.95, 95%CI 1.18−3.23) groups. Similar findings were found when Prostant in combination with antibiotics or hyperthermia compared to the antibiotics (RR 1.78, 95% CI 1.10−2.89) or hyperthermia (RR 1.72, 95% CI 1.23−2.40) alone. However, no significant difference was found between Prostant and α-blockers or hyperthermia therapy in the number of cured patients. Results from single studies or meta-analyses in improving clinical symptoms of CP also showed similar results on effectiveness of Prostant as above, except that hyperthermia seems even better than Prostant on symptoms improvement.

No severe adverse event was reported in all included trials. The main adverse events in Prostant group were reported (in 8 included trials) as diarrhea and anal discomfort.

### 4.2 Overall quality of the evidence

As we mentioned above, GRADE assessment was used for evaluating the quality of the evidence for each primary outcome. Since all the included trials had potential serious risk of bias, level of the evidence should be downgraded. Although majority of the trials had consistent results with good statistical homogeneity (*I*^*2*^ <25%), there are potential clinical heterogeneity which may affect the external validity of the evidence. Furthermore, the number of t trials included in each meta-analysis was small, so the imprecision of the synthesis results may also be influenced. As a result, we downgraded the level of evidence and did not conduct F=funnel plot analysis. However, considering the comprehensive literature searching, the publication bias is not concerned in this review.

Bias may be introduced in a multiple-intervention study if the decisions regarding data analysis are made after seeing the data. In our review, ten included trials were 3-arm trials, so we regarded both Prostant in combination with drugs and the Prostant single application as “multiple-intervention.” We analyzed the data from the combination group and drug group to assess the add-on effect of Prostant; and compared the data from Prostant group and drug group to evaluate the potential effect of Prostant single application for CP. Since these were 2 independent meta-analyses with different purposes, we re-analyzed the data from these 3-arm trials and treated them as 2 two-arm trials. Although the data from the drug group was used twice, we actually did not increase the weight of the set of data because we did not pool the 2 meta-analyses together.

Overall, the quality of the evidence for each primary outcome was evaluated as “very low” in this review (see Table 2).

**Table 2:**
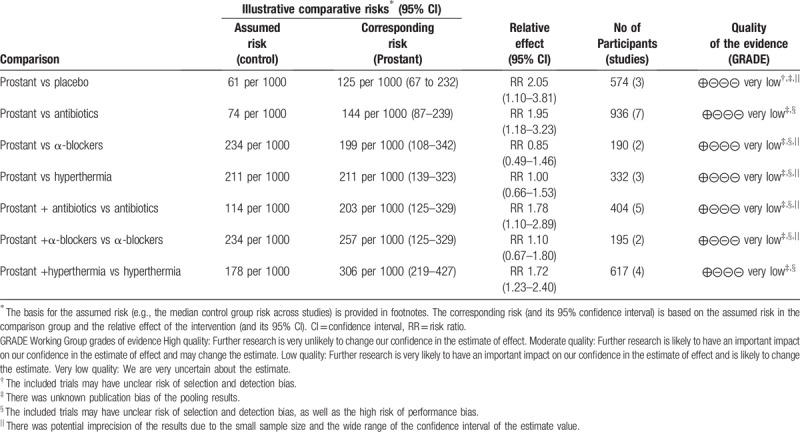
Summary of finding table of different comparisons for the primary outcome: numbers of cured patients.

### 4.3 Implications for the clinical practice

According to this review, Prostant would be recommended as an adjunctive treatment for CP in addition to antibiotics/α-blockers or other nonpharmaceutical therapies. Prostant may help relieve the symptoms and increase the chance of cure on the basis of other treatment. However, due to the limited number of the included trials, it is hard to evaluate the effectiveness of Prostant for different types of CP.

Regarding to the hyperthermia therapy, our finding is similar to the results from one Cochrane review.^[33]^ In our review, hyperthermia therapy was superior to Prostant in improving the symptoms of CP. In the precious Cochrane review^[33]^ assessing the effect and safety of all kinds of nonpharmaceutical treatment for CP included 2 trials with transrectal thermotherapy (237 participants). Based on short-term follow-up, low-quality evidence showed transrectal thermotherapy alone or in combination with medical therapy may decrease prostatitis symptoms slightly when compared with medical therapy alone (NIH-CPSI score MD −2.50, 95% CI −3.82 to −1.18). Transrectal thermotherapy is also a kind of hyperthermia therapy. This suggests that hyperthermia, one nonpharmaceutical therapy, may be an option for patients with CP, especially for those who do not show a marked improvement after drug treatment.

### 4.4 Implications for future researches

Level of the evidence summarized in this review was downgraded mainly due to the poor quality of the included trials. Future researches should pay more attentions to controlling bias during the study period, especially randomization, blinding and missing data management. On the other hand, all the included trials reported cure rate and/or total effective rate as the outcome measurement. Some trials only reported the cure rate. As we mentioned in Section 3, 17 of the included trials reported the cure rate, but only 8 trials reported the NIH-CPSI scores. This may partly explain why the meta-analysis found Prostant was superior in increasing the number of cured patients, but not in reducing the NIH-CPSI scores. Small sample studies may not easily detect differences between groups because of their low statistical efficiency. We recommended that objective outcomes such as NIH-CPSI scores, and EPS-WBC should be used in future researches to measure the effect of intervention for CP.

Furthermore, safety issue is also important for application of the Chinese herbal patent. Future studies should aware of this and report the safety outcome.

## 5 Conclusions

Only very low-quality evidence showed that the Prostant may have some add-on effects on increasing the number of cured patients, relieving pain, and improving the quality of life in patients with CP. The potential benefits of Prostant as adjunctive therapy for CP need to be confirmed in future trials using rigorous methodology. There is insufficient evidence to determine the effectiveness and safety of Prostant for CP when compared with placebo, antibiotics, α-blockers or the hyperthermia therapy.

## Acknowledgments

The authors would like to thank Dr. Guoyan Yang from Western Sydney University for her modifying and polishing the language of this article.

## Author contributions

Wang B and Li HS conceived the research topic. Cao HJ, Liang SB and Liu JP formulated the plan of the study. Liang SB, Wang JS, Guan SQ, Zhu YS and Dai HH screened the literatures and selected the eligible trials according to the above criteria. Disagreements were solved by discussion with Cao HJ. Cao HJ and Liang SB performed the statistical analysis and drafted the manuscript. Liu JP helped to draft the protocol and the final manuscript. Wang B and Li HS participated in the study design and coordination. Zhou CY participated in drafting and revising the manuscript. All authors read and approved the final manuscript.

**Conceptualization:** Wang Bin, Li Hai-song.

**Data curation:** Cao Hui-juan, Liang Shi-bing, Liu Jian-ping, Wang Ji-sheng, Guan Si-qi, Zhu Yu-tian, Dai Heng-heng.

**Formal analysis:** Cao Hui-juan, Liang Shi-bing, Liu Jian-ping, Zhu Yu-tian, Zhou Chun-yu.

**Funding acquisition:** Cao Hui-juan, Wang Bin, Li Hai-song.

**Investigation:** Cao Hui-juan, Liang Shi-bing.

**Methodology:** Cao Hui-juan, Liang Shi-bing, Liu Jian-ping.

**Project administration:** Wang Bin, Li Hai-song.

**Resources:** Cao Hui-juan, Liang Shi-bing, Liu Jian-ping, Wang Ji-sheng, Guan Si-qi, Dai Heng-heng, Zhou Chun-yu.

**Software:** Cao Hui-juan, Liang Shi-bing, Liu Jian-ping, Wang Ji-sheng, Guan Si-qi, Zhu Yu-tian, Dai Heng-heng, Zhou Chun-yu.

**Supervision:** Cao Hui-juan.

**Writing – Original Draft:** Cao Hui-juan, Liang Shi-bing, Wang Bin, Li Hai-song, Wang Ji-sheng, Guan Si-qi, Zhu Yu-tian, Dai Heng-heng, Zhou Chun-yu.

**Writing – Review & Editing:** Cao Hui-juan, Liang Shi-bing, Liu Jian-ping.

Liang Shi-bing orcid: 0000-0002-3780-5107.
